# Supplementary material for: The shape of the contact–density function matters when modelling parasite transmission in fluctuating populations
Source: R Soc Open Sci. 2017 Nov 22;4(11):171308. doi: 10.1098/rsos.171308 (PMC5717690; doi:10.1098/rsos.171308)
Supplement: Supporting Information to The shape of the contact-density function matters when modelling disease transmission in fluctuating populations [file rsos171308supp1.pdf]

Supplementary information

The shape of the contact-density function matters when modelling parasite transmission in fluctuating populations

Benny Borremans[1,2,*], Jonas Reijniers[1,3], Niel Hens[4,5], Herwig Leirs[1]

Affiliations:

[1] Evolutionary Ecology Group, University of Antwerp, Belgium

[2] Department of Ecology and Evolutionary Biology, University of California Los Angeles, United States

[3] Department of Engineering Management, University of Antwerp, Belgium

[4] Centre for Health Economics Research & Modelling Infectious Diseases (CHERMID – VAXINFECTIO), University of Antwerp, Belgium

[5] Interuniversity Institute for Biostatistics and Statistical Bioinformatics (I-BIOSTAT), Hasselt University, Belgium

* Correspondence: bennyborremans@gmail.com

Contents

1. Seasonal population dynamics 2
2. Timing of introduction 2
3. Fitting q_i 5
4. Transmission rate 7
5. References 9

1. Seasonal population dynamics

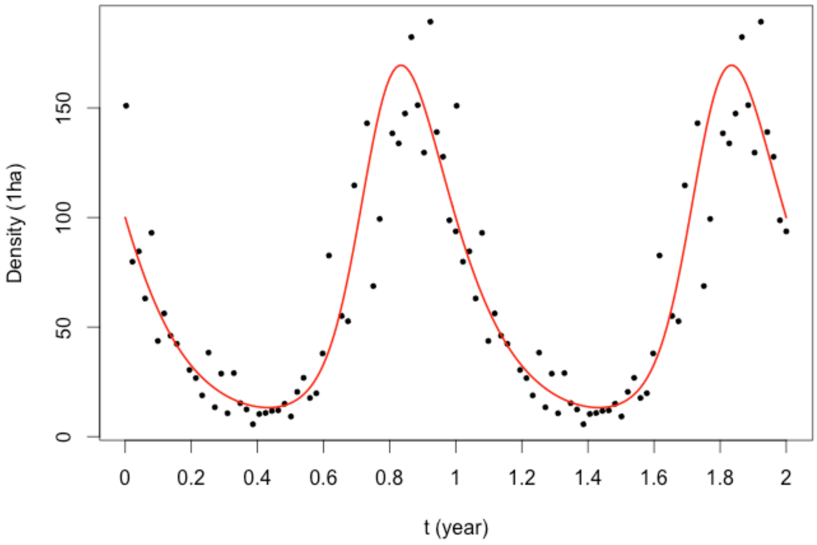

Figure S1-1. Demographic model (red line) fitted to field data (black dots = mean population density) spanning 20 years. Fitted parameters: s = 2.7, φ = 0.42, μ = 7. The birth function $B(t) = k \exp\left[-s \cos^2(\pi t - \varphi)\right]$ as described in the main text and in Peel *et al.* [1].

2. Timing of introduction

Because of seasonal birth pulses and fluctuating densities, the time at which the infection is introduced into the population (I→1) may affect transmission dynamics and infection/persistence probabilities. To investigate this effect, the models were run for a range of introduction times.

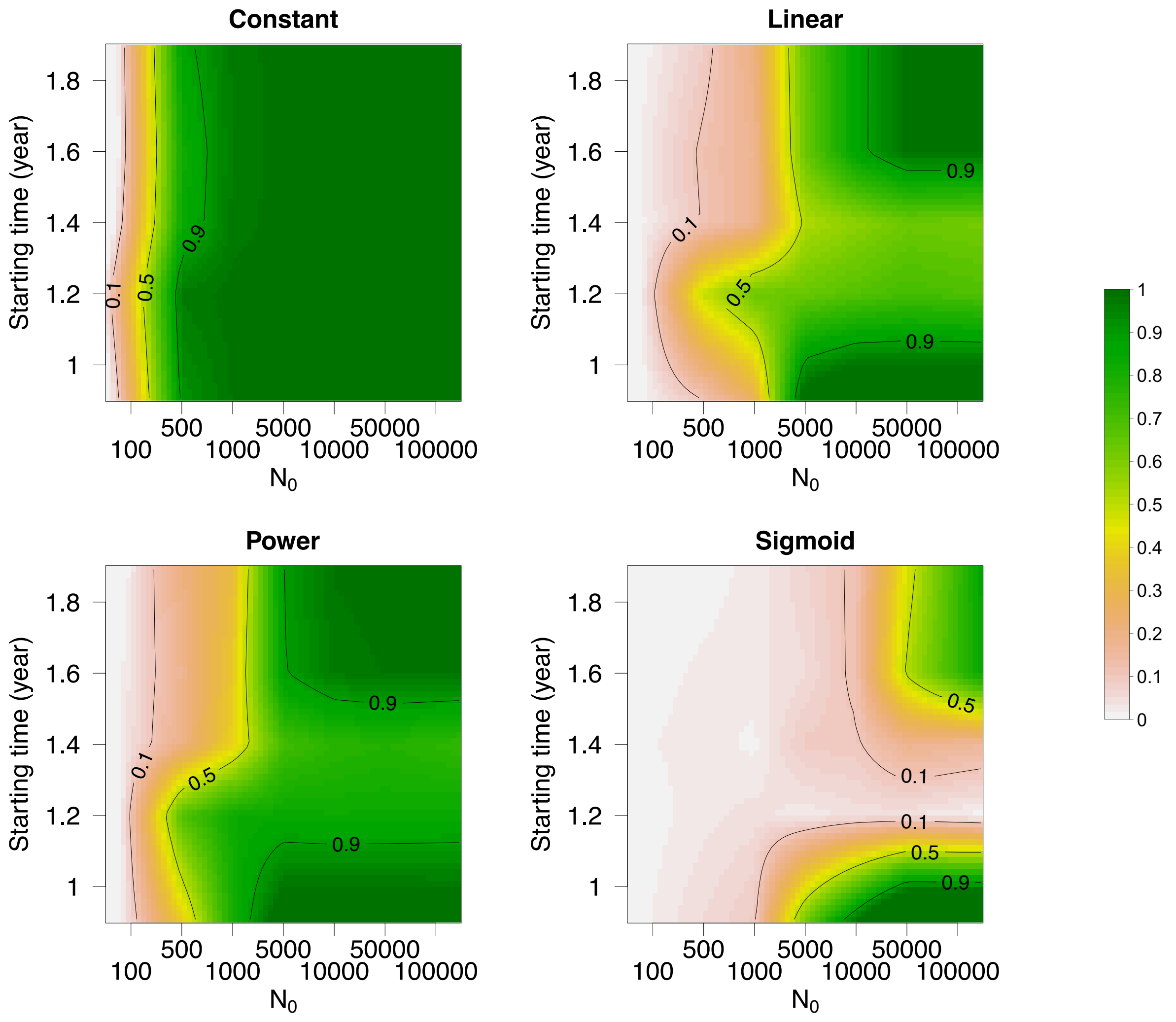

Figure S2-1. Invasion probability for the different contact-density functions, for a range of infection introduction times t_0 and initial population sizes N_0. Transmission rate p = 50, infectious period $1/\gamma$ = 30 days. Simulations were conducted for all values indicated by tick marks on the axes, and results are interpolated between these values for illustration.

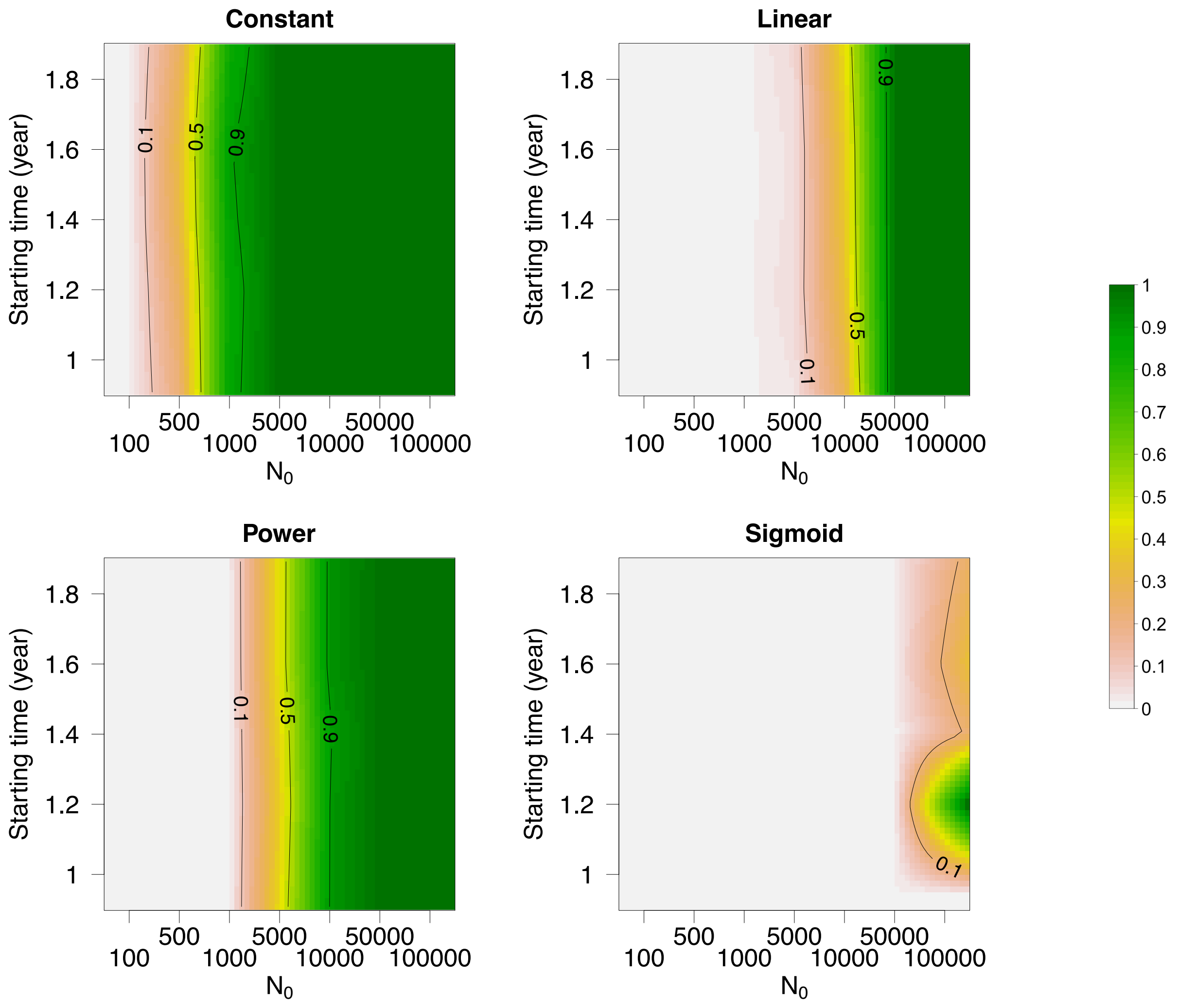

Figure S2-2. Persistence probability for the different contact-density functions, for a range of infection introduction times t_0 and initial population sizes N_0. Transmission rate p = 50, infectious period $1/\gamma$ = 30 days. Simulations were conducted for all values indicated by tick marks on the axes, and results are interpolated between these values for illustration.

3. Fitting q_i

In order to correctly compare the different functions, the transmission parameter β must be fitted to a certain result, just like a model must be fitted to real data. In the SIR models, the rate of infection is written as $\beta S\frac{I}{N}$, where $\beta = q_i p c_i$, c_i is contact-density function i, p the transmission rate (which is the same for every contact-density function) and q_i, a fitting constant that is specific for each contact-density function i and ensures that a certain model result or parameter is equal for each function. Although there are numerous parameters to choose from for fitting q_i, three methods were chosen in this study. Method one, which was used to generate the results in the main text, fits q_i so that $\beta = q_i \times \sum_{j=1}^{300} f_c(\frac{N}{A})_j \times h(\frac{N}{A})_j$. In method two, q_i was fitted to ensure that the maximum prevalence (I/N) was 40%. Method three fitted q_i so that the annual cumulative incidence was 200% of the starting population (i.e. at the end of a year, $2N_0$ infections occurred). Table S3-1 shows the different q_i values, and the resulting β-density functions can be seen in Figure S3-1.

Table S3-1. Fitting constants q_i for each function i, using three different fitting methods.

Function		q_i (β1)	q_i (β2)	q_i (β3)
Constant	$1.14\,(\frac{N}{A})^0$	1.00	1.49	0.64
Linear	$0.0092\,\frac{N}{A}$	1.82	0.86	1.06
Power	$0.124(\frac{N}{A})^{0.5}$	1.22	0.7	0.73
Sigmoid	$2.13/(1+e^{-0.05\,((\frac{N}{A})-101.2)}$	1.77	0.71	0.99

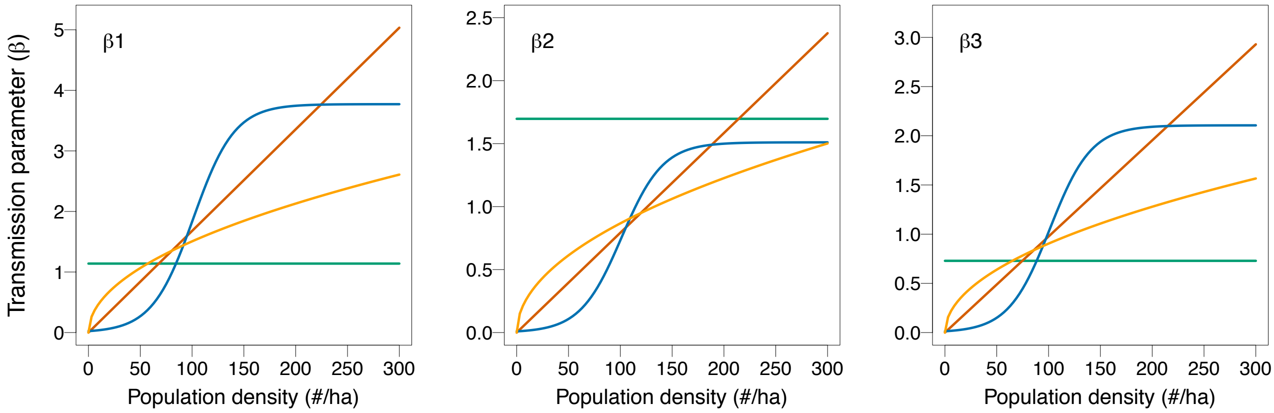

Figure S3-1. Different β-density functions resulting from different fitting parameters q, where $\beta = c_i \times p \times q_i$. $\beta1$: using q_1, fitted so that β integrated across one year is the same for each function; $\beta2$: using q_2, fitted so that the maximum annual prevalence is 40%; $\beta3$: using q_3, fitted so that the annual cumulative incidence is 200% of the starting population ($2N_0$). Note that the Y-axis scales are different. Green = constant, red = linear, yellow = power, blue = sigmoid.

4. Transmission rate

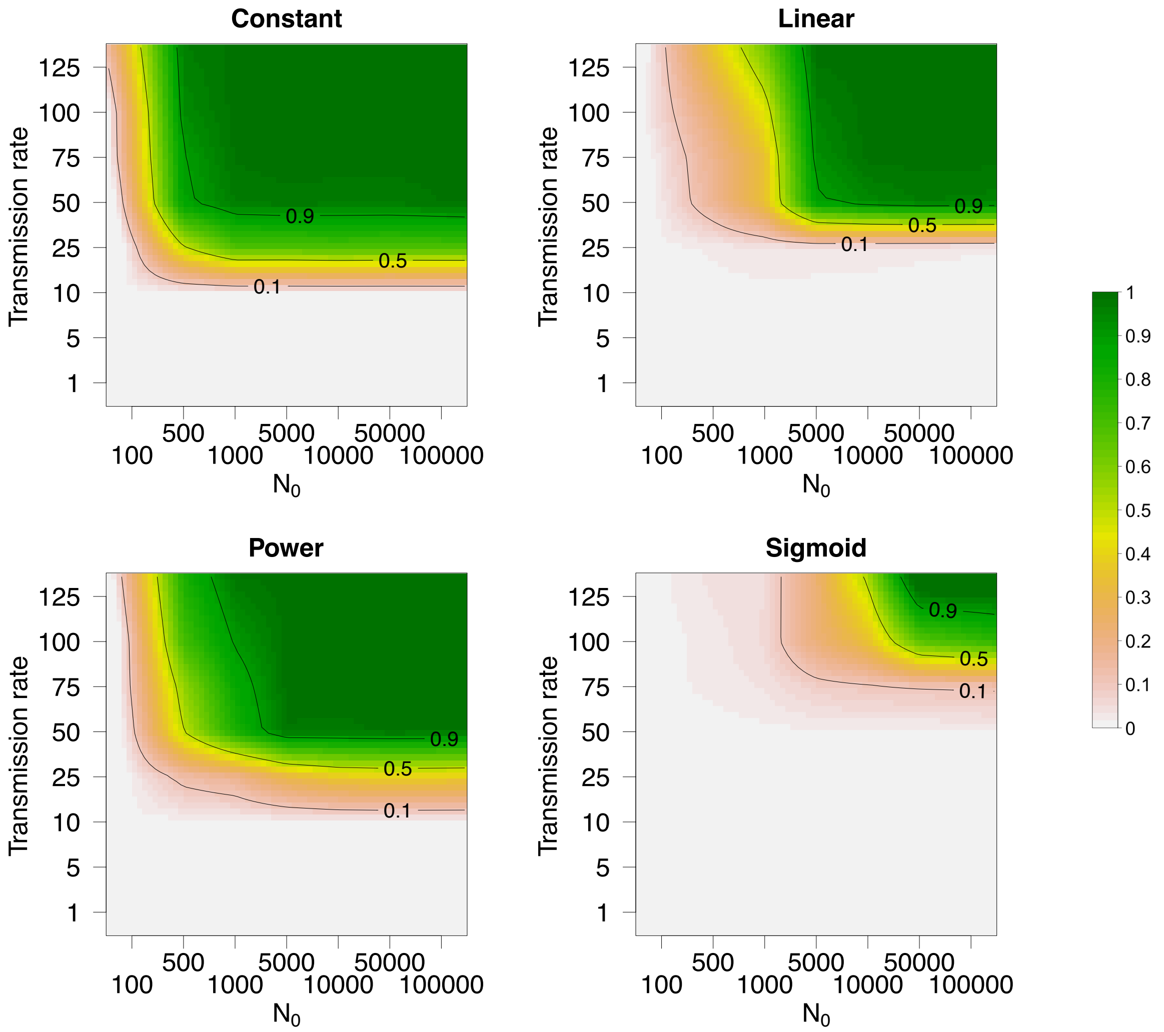

Figure S4-1. Invasion probability for the different contact-density functions, for a range of transmission rates *p* (before multiplication with each function's β-fitting constant q_i) and initial population sizes N_0 (infectious period $1/\gamma = 30$ days), using β-fitting method 1 (β integrated across one year is the same for each function).

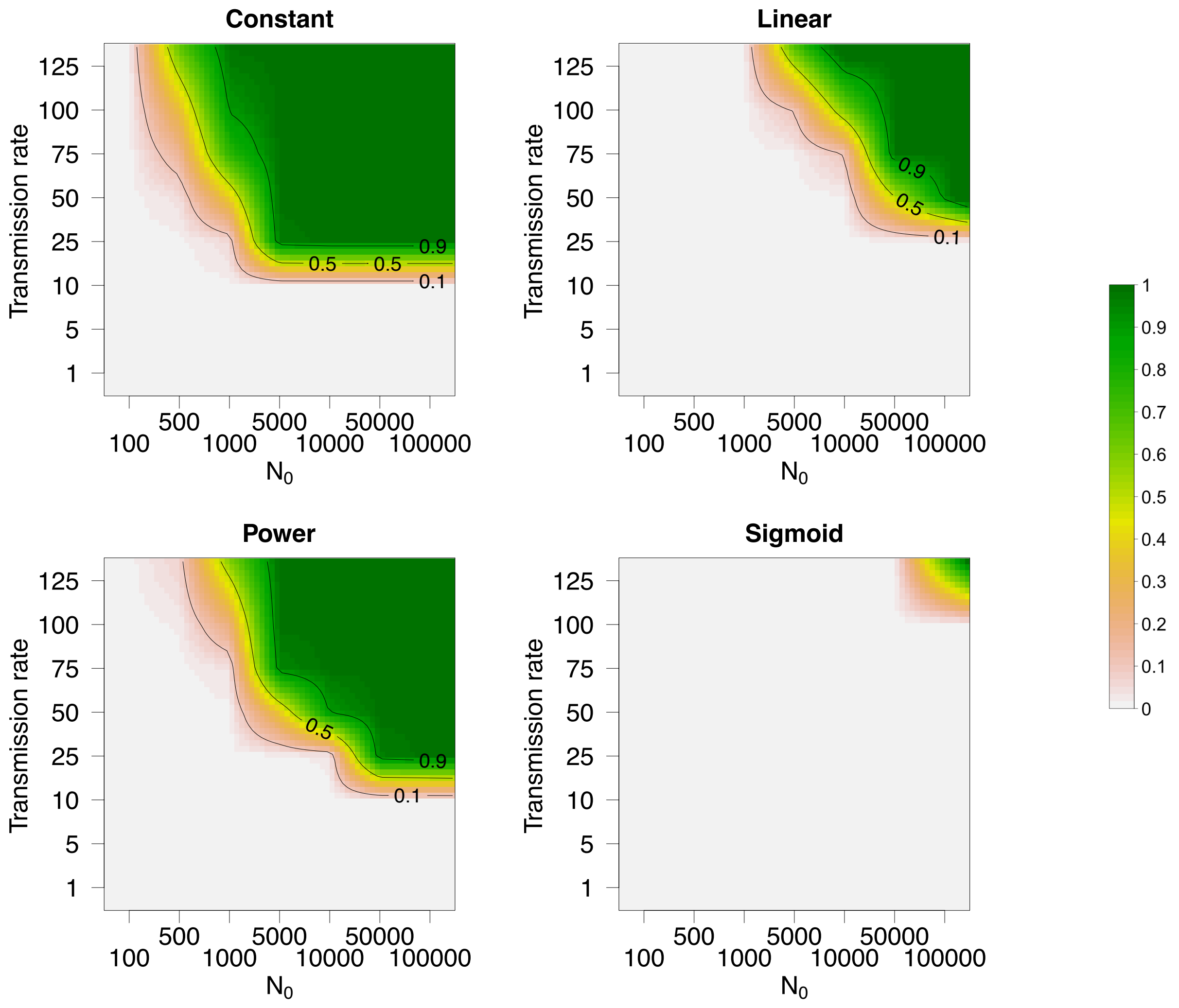

Figure S4-2. Persistence probabilities for the different contact-density functions, for a range of transmission rates p (before multiplication with each function's β-fitting constant q_i) and initial population sizes N_0 (infectious period $1/\gamma$ = 30 days), using β-fitting method 1 (β integrated across one year is the same for each function).

5. References

1. Peel, A. J., Pulliam, J. R. C., Luis, A. D., Plowright, R. K., Shea, T. J. O., Hayman, D. T. S., Wood, J. L. N., Webb, C. T. & Restif, O. 2014 The effect of seasonal birth pulses on pathogen persistence in wild mammal populations. *Proc. R. Soc. B Biol. Sci.* **281**, 20132962. (doi:10.1098/rspb.2013.2962)
